# Wild capuchin monkeys adjust stone tools according to changing nut properties

**DOI:** 10.1038/srep33089

**Published:** 2016-09-14

**Authors:** Lydia V. Luncz, Tiago Falótico, Alejandra Pascual-Garrido, Clara Corat, Hannah Mosley, Michael Haslam

**Affiliations:** 1 School of Archaeology, University of Oxford, UK; 2 Institute of Psychology, University of São Paulo, Brazil

## Abstract

Animals foraging in their natural environments need to be proficient at recognizing and responding to changes in food targets that affect accessibility or pose a risk. Wild bearded capuchin monkeys (*Sapajus libidinosus*) use stone tools to access a variety of nut species, including otherwise inaccessible foods. This study tests whether wild capuchins from Serra da Capivara National Park in Brazil adjust their tool selection when processing cashew (*Anacardium* spp.) nuts. During the ripening process of cashew nuts, the amount of caustic defensive substance in the nut mesocarp decreases. We conducted field experiments to test whether capuchins adapt their stone hammer selection to changing properties of the target nut, using stones of different weights and two maturation stages of cashew nuts. The results show that although fresh nuts are easier to crack, capuchin monkeys used larger stone tools to open them, which may help the monkeys avoid contact with the caustic hazard in fresh nuts. We demonstrate that capuchin monkeys are actively able to distinguish between the maturation stages within one nut species, and to adapt their foraging behaviour accordingly.

When foraging in their natural environments, animals need to recognize and respond to changes in food targets. This ability is especially useful when dealing with a defensive mechanism of the target food (e.g., toxicity, venom, irritants) where risk of injury represents an important cost for the forager^1^. Several animal species have evolved foraging strategies to minimize those potential risks involved when dealing with dangerous prey. For example, meerkats (*Suricata suricatta*) are able to effectively disarm scorpions to reduce the threat of injury to younger group members^2^. Some animals also use foraging strategies that include objects to reduce or prevent the risk of injury involved in consuming challenging food sources. Bottlenose dolphins (*Tursiops* sp.), for example, use detached marine sponges over their nose to nuzzle for prey in rocky sea grounds^3^. Primates have especially been shown to exhibit a variety of solutions as a response to dealing with the defense mechanisms of harmful target foods. White-faced capuchins (*Cebus capucinus*) wrap naturally-defended caterpillars and fruits in leaves before rubbing them against a substrate, which is suggested to be a means of avoiding noxious substances^4^. Chimpanzees (*Pan troglodytes*) foraging on aggressive army ant nests use nearby trees to reposition themselves off the ground, from where they can more securely dip for the ants below^5^. Similarly, chimpanzees are able to minimize risk to accompanying young by predating on aggressive army ants on ant trails rather than at the ant nests, even though feeding at nests yields a higher rate of energetic return^6^. Different solutions to minimize painful bites when preying on army ants, including stick tool use, are described for multiple chimpanzee populations throughout Africa^6^,^7^. The observed diversity suggests that hazard avoidance may be a socially influenced response^8^.

Animal tool use increases net gain by enabling the exploitation of inaccessible or costly to process food resources. This allows access to higher nutritional value foods^9^,^10^, an adaptive advantage in times of food scarcity^11^,^12^, competition^13^, or opportunistic foraging^14^. Tool size, weight and required transport distance influence the amount of energy expended during a given task^15^,^16^,^17^. To maintain the balance between cost and gain, individuals must recognize and manage energy expenditure relative to the task at hand. This requires a comprehension of the functional aspects of the food item, its physical constraints and potential risks involved.

Selectivity in the physical properties of tools has been observed amongst different primate species^18^,^19^,^20^ as well as corvids^21^. Wild chimpanzees adjust their tool selection to changing properties within one target food^22^,^23^,^24^. For example, with increasing ripeness of *Coula edulis* nuts within one fruiting season the nuts become easier to crack and chimpanzees adjust their tool selection accordingly. Neighboring chimpanzee communities that live in similar environmental conditions however react differently to changes in the target food. This indicates that responses to changing food items are socially learned^22^,^24^. Reports on wild capuchin monkeys (genera *Cebus* and *Sapajus*) suggest that they are capable of both hazard reduction and adjustment of their behaviour to match food properties^4^,^25^. When foraging for embedded larvae (*S. apella*) capuchins are able to distinguish between pay-off rates between two stages of the same foraging substrates^26^. Bearded capuchins (*Sapajus libidinosus*) have been observed modifying the force - although not the stone tool - throughout the sequence of cracking a single nut as a response to the state of the nut^27^. The same monkeys selected different stone tool sizes based on the resistance of different nut species. It is important to note that no primate studies to date have been able to determine whether a wild animal considers two states of the same food species (e.g., fresh or dry nuts, intact or partially-opened nuts) to be two different foods. Instead, these studies concentrate on animal behaviour towards the differing food targets, and we follow the same approach here.

Capuchins in Serra da Capivara National Park (SCNP) are known to exhibit a range of tool using behaviour, including different stick and stone tools for foraging, social display and self-maintenance^12^,^28^,^29^,^30^,^31^. They habitually use stone tools to crack open cashew (*Anacardium* spp.) nuts, which are native to the northeastern part of Brazil^32^. Cashew trees produce a pseudo-fruit in the form of an apple and a hard nut at the end of the apple which holds the reproductive seed^25^. The shell of unprocessed cashew nuts contains caustic Cashew Nut Shell Liquid (CNSL), a phenolic resin (similar to poison ivy or poison oak), which causes severe reactions when in contact with the skin and mucosa. We focused on capuchin processing of cashew nuts because, as cashews ripen, they appeared to change in both nut hardness and in the amount of CNSL in the nut mesocarp.

At SCNP, capuchin monkeys use stone tools to open all maturity stages of cashew nuts. Average weight of tools used by the monkeys to open cashews was greater than the average of available stones in the area, indicating a selecting behaviour^32^. On average, males use stone tools more frequently than females to process cashew nuts (70% of the episodes), due to the larger body size of males this is a common pattern in capuchins populations^33^,^34^,^35^. There is no difference in the weight, size and success of stone tool use between sexes at SCNP, however juveniles are less successful than adults in opening cashews^32^. Even though fresh cashew nuts are on average 25% larger than dry ones, tools are not necessary to open fresh nuts as the outer mesocarp is still soft. Some monkeys in SCNP have been observed to bite and rip open fresh cashews using their hands and teeth. However, adult group members usually used stone tools to open both fresh and dry cashew nuts, before extracting the cashew kernel with either fingers or teeth.

Our focus on tool-based processing of this potentially hazardous foraging item is of additional interest, as a different capuchin group of the same species at the Fazenda Boa Vista (FBV) site, approximately 350 km away from Serra da Capivara, specifically avoids contact with CNSL when eating cashew nuts^25^,^36^. At FBV the monkeys have never been seen using stone tools to open fresh cashews nuts. Instead they use a rubbing technique which allows them to extract the kernel using their fingers to avoid contact with the CNSL^25^,^36^. The FBV capuchins use stone tools only to process dry nuts towards the end of the fruiting season, when the toxic CNSL hardens into a more resinous material that is less likely to come into contact with the monkey’s skin.

Based on previous studies, we hypothesized that wild capuchin monkeys would adapt their tool selection when processing a food item that changes its condition over time. To test this hypothesis, we carried out field experiments with a group of wild bearded capuchins in Serra da Capivara National Park (SCNP), northeast Brazil.

As part of this study, we predicted (Table 1) that cashew nuts would increase in hardness as they dry and mature, which would require a correspondingly greater force from the monkeys to open dry nuts. We tested cashew nut hardness of different maturity stages using a standardized nut cracking device. We also expected to find that fresh cashew nuts contain more easily dispersed CSNL than dry nuts, as it is in a more liquid form in this earlier maturation stage. We therefore compared exposed CSNL between the different cashew maturation stages. The capuchins were therefore expected to use fewer strikes to open a nut when using heavier stones, and were expected to use heavier stones to open dry (harder) nuts.

## Methods

### The field site

Serra da Capivara National Park is located in the northeast region of Brazil, a region of dry thorn-bush vegetation (‘Caatinga’), with a highly seasonal semi-arid climate^37^. Research was carried out with the fully habituated Pedra Furada (PF) group^32^, which inhabits the southeast of the park (8°50’S, 42°33’W). At the time of the study this group had 34–35 individuals (Supplementary Table S1). All cashew nuts used in this study were obtained from a commercial cashew farm in São Raimundo Nonato, Piauí, located next to SCNP, to allow the selection of sufficient nuts of differing ripeness stages. Any pseudo-fruits were removed prior to the experiments, to replicate the condition of nuts on which the SCNP capuchins use stone tools under natural circumstances.

### Physical differences between ripeness stages of cashew nuts

**Figure 1:**
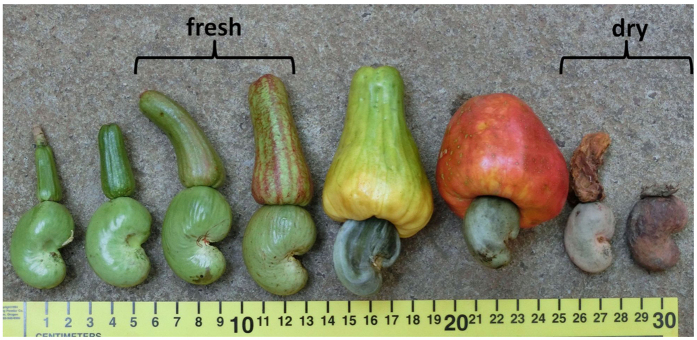
Progressive ripeness stages of cashew nuts. For the experiments we used the stages “fresh” and “dry”.

To test for physical differences in nut properties, manual nut-cracking experiments were carried out. This study involved dropping a fixed weight of 350 g repeatedly onto a nut from a height of 30 cm until the nut broke open. The number of hits needed to break open 40 individual cashew nuts of each of the two ripeness stages (fresh and dry, Fig. 1) were counted. A fresh cashew nut is from the early stage of fruit ripening, and is bright green in color with an underdeveloped pseudo fruit (the apple) attached to it. At this stage the nut’s outer shell is rubbery and its interior is spongy. In the manual nut-cracking test the nut was defined as opened when it showed the first rip in the outer shell. Dry nuts from the later ripening stages are more brittle, usually grey to brown in colour and when successfully struck crack open to reveal the inner kernel. To compare the number of hits needed to successfully open the fresh and dry cashew nut samples, we used a Mann Whitney U-test.

In order to measure the amount of CNSL exposed in the process of opening the nuts, the manual nut-cracking experiment was performed on water resistant, gridded paper. After the nut was successfully opened the exposed CNSL was manually spread across paper. The maximum liquid distribution was then digitized by taking a high definition photograph, and the total area calculated using Photoshop CS6. A Mann Whitney U-test was used to compare the areas covered with CNSL fresh and dry cashew nuts.

### Field experiments on tool selection

**Figure 2:**
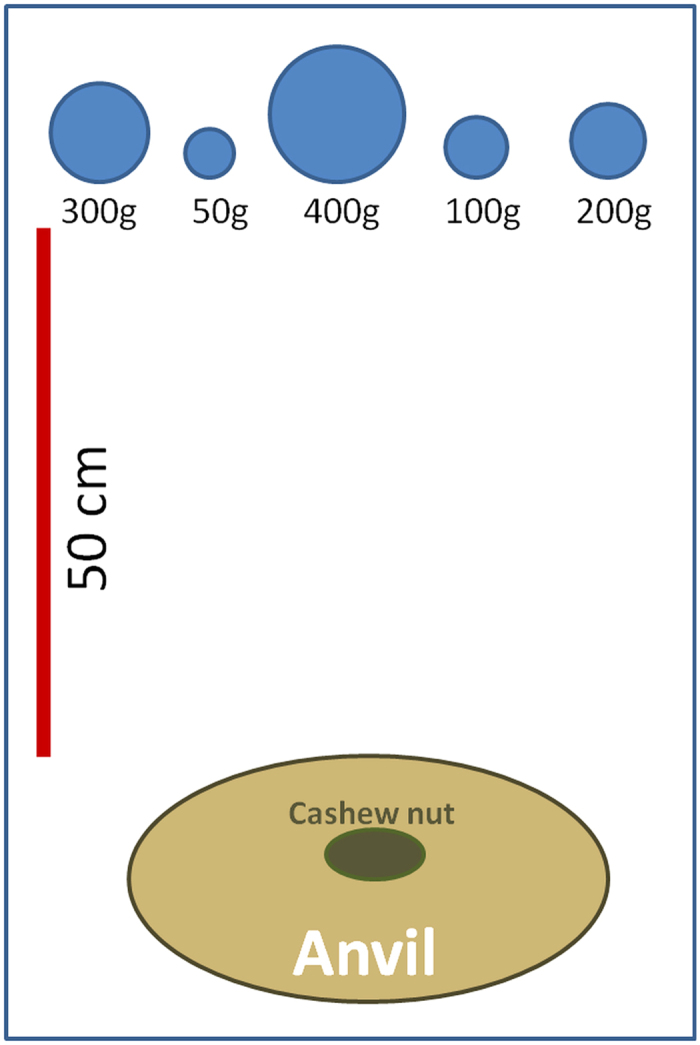
Experimental set-up. Five quartzite stones of different weights were presented in random order to the monkeys at a sandstone anvil, which was placed 50 cm from the tools. In the cashew season of 2013 we tested capuchin tool selection for opening dry cashews, in 2014 we tested the selection when opening fresh cashew nuts.

To assess capuchin selection for stone hammer weight, we conducted controlled field experiments (Supplementary Video 1). Stones in five weight categories — 50 g, 100 g, 200 g, 300 g and 400 g — were presented to the monkeys, with stone weights varying ± 10% around these values in each category. All stones included in the experiments were of similar oval shape (ratio: mean length/mean width of the 5 weight categories: 1.29; 1.39; 1.26; 1.32; 1.29). At the start of each trial the stones were placed 50 cm from a natural sandstone anvil, on which a cashew nut was placed (Fig. 2). All pounding tools were quartzite, the preferred hammer material used by the SCNP capuchins, gathered from the immediate vicinity of the experiment (part of the group’s natural foraging range). Provided tools where all of the same material to ensure that increasing stone weight correlated with increasing stone size. The correlation between tool size (length, width and thickness) and weight was further tested using a Pearson’s rank correlation test. The order in which the weight categories were presented was randomized for every trial. Once used, a tool was replaced with an unused one to avoid any possible influence of previously used stones.

All successful experimental trials were fully documented using a hand held Canon video camera. Only the first tool selection of each trial was considered in the analyses. The number of strikes required to open each nut was extracted from the video record. If an individual did not select a tool, but took the nut to another location, or another adult individual was competing over the nut, the trial was discarded. The experiment was conducted in two periods. In May 2013, 15 adults (5 adult males, 10 adult females) were tested with dry cashew nuts (n = 270 trials). In September 2014, 14 adults (5 males and 9 females) were tested with fresh cashew nuts (n = 182 trials). Individuals were only included in the analysis when at least eight independent trials were recorded.

To investigate whether the weight of the tool influenced the number of hits needed to crack open nuts of differing ripeness, we used a GLMM^38^ with poisson error structure, with the response variable being the number of strikes applied. As predictor variables the ripeness stage of the cashew, the sex of the individual and the selected tool weight were included. Animal ID was controlled for by including it as a random effect into the model. Type I error rates were kept at the nominal level of 5% by including random slopes of tool selection within individuals into the model^39^,^40^. The null model did not comprise the variable ripeness stage of the cashew and the selected weight.

In order to test whether capuchin tool selection differs according to the ripeness stage of the target cashew nut, we used a GLMM with poisson error structure, with the selected tool weight set as the response variable and the ripeness stage of the cashew, sex of the individual as predictor variables and animal ID as random effect. Additionally, random slopes of tool selection within individuals (see above) were included into the model. The null model did not comprise the predictor variables. A poisson error structure was modelled because the distribution of the response clearly resembled such a distribution much more than a Gaussian distribution (and using a Gaussian error distribution was likely to reveal problems regarding assumptions about the residuals).

Both models were fitted in R^41^ using the function lmer of the R-package lme4^42^. The significance of the comparison between the full and the null models was established using a likelihood ratio test (R function anova with the argument set to “Chisq”^40^,^43^; p-values for the individual effect of the included variables were established by likelihood ratio tests comparing the full with the respective reduced model, excluding the variable in question)^39^. The assumptions of normally distributed and homogeneous residuals were fulfilled by visually inspecting a qqplot and the model stability was tested by excluding one individual at the time from the data, with no influential individual found. Both models revealed no overdispersion. After eliminating random effects, Variance Inflation Factors were tested using the R-package car^44^ and no indication for collinearity was found.

### Ethical note

The research was approved by IBAMA/ICMBio (authorizations 37615-1), and MCT/CNPq 000482/2013-7, adhered to the ASAB/ABS Guidelines for the Use of Animals in Research, and followed all ethical guidelines for animal research of the Institute of Psychology-USP.

## Results

### Physical differences between ripeness stages

**Figure 3:**
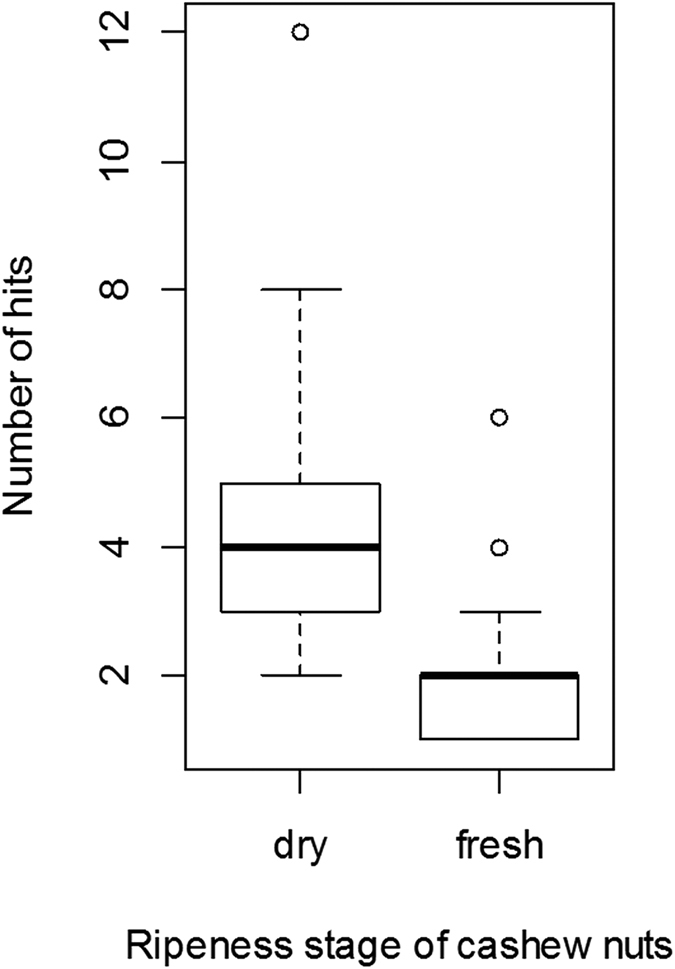
Differences in durability between two different stages of cashew nut ripeness. The y-axis shows the number of hits needed to crack open either a dry or a fresh nut with a standardized nut cracking device. The boxplot shows the upper and lower quartile with the median presented by the thicker horizontal line. The whiskers present the maximum and minimum range of the data (excluding outliers). The circles show individual outliers.

**Figure 4:**
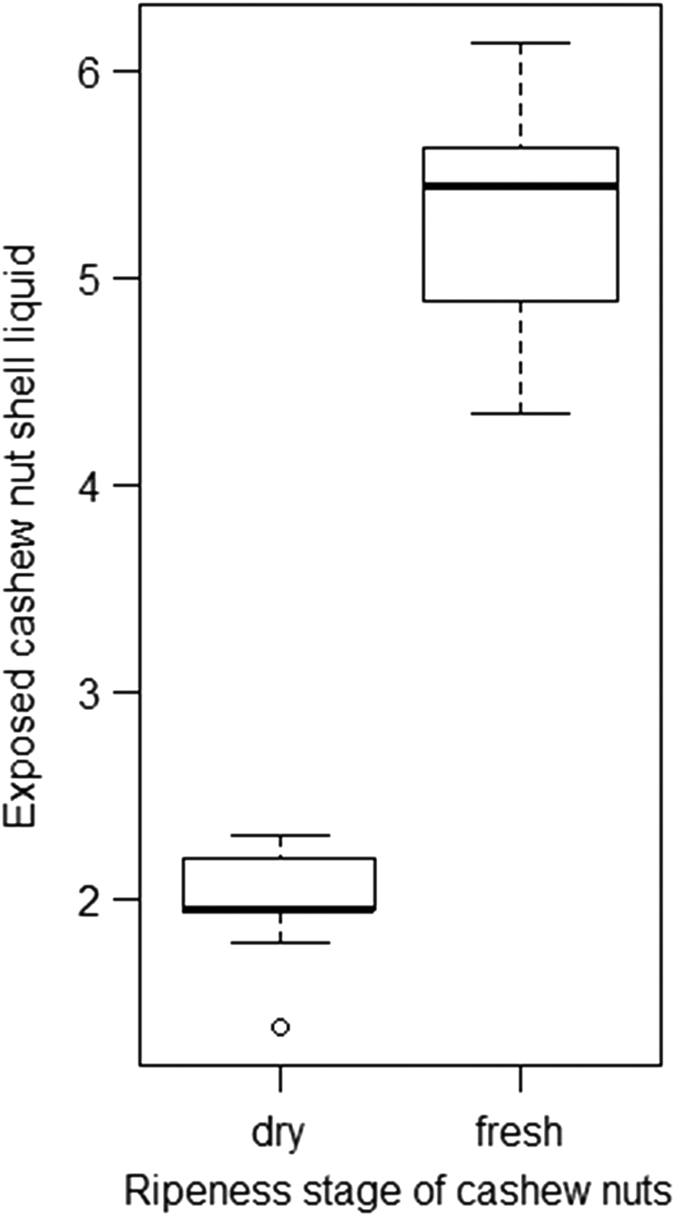
Differences in CNSL exposure of two different ripeness stages of cashew nuts. The amount of CNSL exposed during nut cracking experiments (carried out by human experimenters) is displayed on the y-axis for either dry or fresh nuts. The boxplot shows the upper and lower quartile with the median presented by the thicker horizontal line. The whiskers present the maximum and minimum range of the data (excluding outliers). The circle shows an individual outlier.

Our results indicate that physical properties of cashew nuts change during the ripening process. As predicted, dry nuts are harder, and therefore require more hits to be cracked open than fresh nuts (Mann Whitney U-test: U = 174.5, N = 80, p-value < 0.001). On average fresh nuts in our manual tests were found to open with only half the number of hits required for dry nuts (Fig. 3). Additionally, fresh nuts contained significantly more readily-dispersed CNSL than dry nuts (Mann Whitney U-test: U = 0.0, N = 30, p < 0.001), (Fig. 4).

### Tool selection when cracking nuts

**Figure 5:**
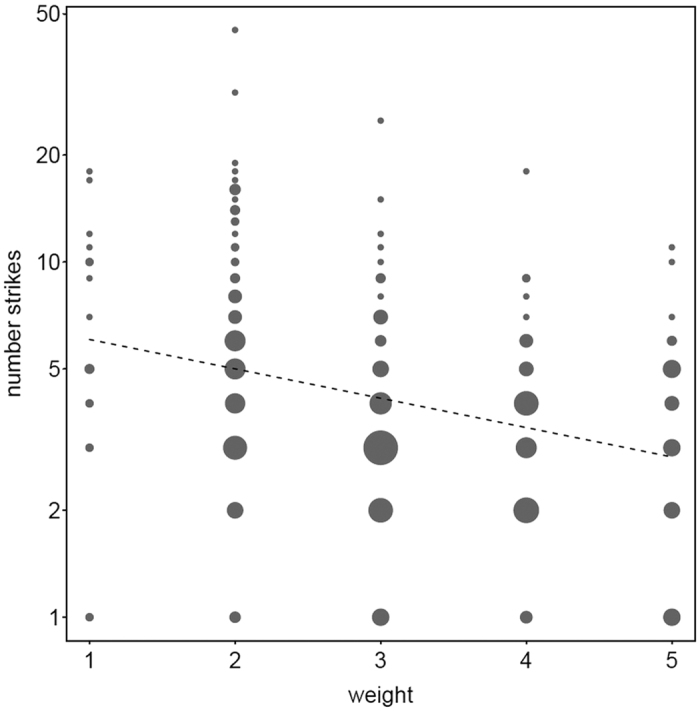
The influence of tool weight on nut cracking efficiency. The number of strikes the capuchins used to open both fresh and dry cashew nuts decreases with the increase of stone tool weight.

Tool weight significantly affected the number of strikes that capuchins needed to open a nut. The comparison between the null and full model revealed clear significance (GLMM: N = 411, χ^2^ = 16.2, df = 2, P < 0.001). The monkeys used fewer strikes with increasing tool weight (GLMM: N = 411, χ^2^ = 28.5, df = 2, P < 0.001) (Fig. 5). This finding held true regardless of the progressing ripeness stage of the nut (P = 0.49) and the effect was the same for males and females (P = 0.22) (Supplementary Table S2).

**Figure 6:**
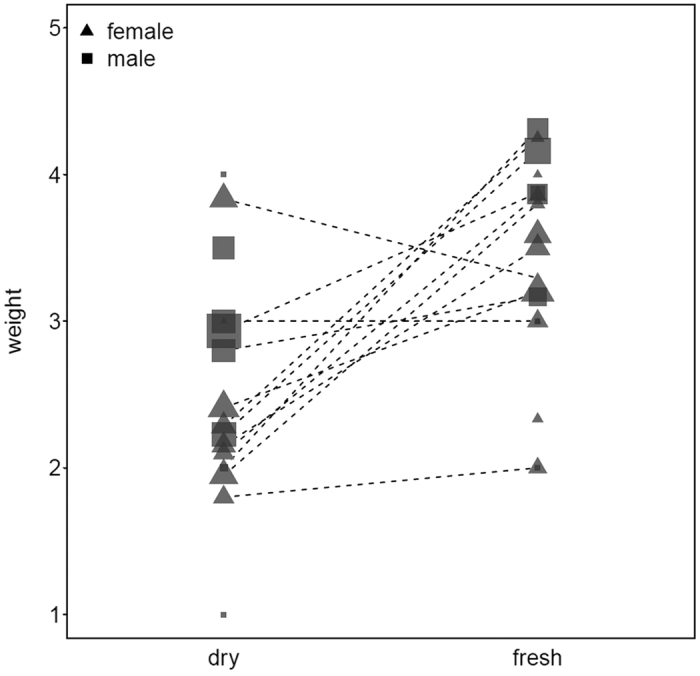
Capuchin tool selection (by weight) at different cashew nut ripeness stages. Size of the symbol is relative to the number of trials for that individual. The dotted lines connect the same individual (for dry and hard cashew nuts).

Capuchin monkeys adjusted their tool selection to the maturation stage of cashew nuts. The comparison of the null to the full model revealed clear significance (GLMM: N = 411, χ^2^ = 20.08, df = 2, P < 0.001) (Fig. 6). When nuts were fresh, monkeys used heavier tools to crack them open compared to cracking dry nuts (GLMM: N = 411, χ^2^ = 18.34, df = 1, P < 0.001). Males also used heavier tools than females (GLMM: N = 411, χ^2^ = 5.97, df = 1, P = 0.01) (Supplementary Table S3). Weight and size of selected stone tools showed a significant relationship (r = 0.98, N = 100, P < 0.001) where heavier tools increasingly presented a larger surface area.

## Discussion

Wild bearded capuchin monkeys at SCNP adjusted their tool selection to different maturation stages within one target food species. The tested individuals used different stone weights for fresh and dry cashew nuts. The results of this study support both our initial predictions (Table 1) concerning cashew nut properties, showing that fresh cashews are easier to crack open than dry cashews, and that fresh nuts release more easily dispersed CSNL. We also found, as expected, that heavier stones allowed the capuchins to open nuts using fewer strikes, regardless of maturation stage of the target nut.

However, contrary to our final prediction, we found that the SCNP capuchins used significantly lighter stones to process the harder dry nuts than the softer fresh nuts. An increase in weight of the tool correlates strongly with an increase in size and therefore surface area of a tool. Taken together, these findings suggest that the capuchins were selecting larger stones and using fewer strikes to open the softer fresh nuts, which release a large amount of CNSL when struck.

We can suggest a number of potential explanations for this unexpected result: (i) capuchin monkeys might consistently fail to identify that harder nuts require more force to open, (ii) capuchin monkeys selected heavier (and therefore larger) stones because the fresh nuts are slightly larger, disregarding nut hardness differences, or (iii) capuchin monkeys use heavier (and therefore larger) stones on fresh nuts as a means of shielding themselves from toxic CSNL released from cashews during processing.

The first of these explanations contradicts numerous results from studies of wild *S. libidinosus* at FBV, where stone selection based on food hardness has been well-documented, and it is therefore unlikely to be valid^19^,^45^. We note that the FBV experiments on stone weight selection have only included non-caustic foods. However, the authors of those studies consider their results on stone size and hardness generalizable across all *S. libidinosus*, and until conclusive evidence to the contrary is presented, this suggestion has not been refuted. The second explanation would also require the SCNP capuchins to not possess the strong tendency to take food hardness into account seen at FBV, and so is again considered here to be implausible.

The final explanation, of capuchins avoiding CSNL exposure through tool selection, is plausible given that a significant decrease in the amount of released CNSL is the primary difference between striking fresh as opposed to dry nuts. Even though fresh cashew nuts are on average larger than dry ones, tools are actually not necessary to open the soft outer mesocarp of fresh cashew nuts. Occasionally, SCNP capuchins were observed to bite or rip open fresh nuts using their hands and teeth, with this behaviour sometimes causing red blistering around the mouth area^32^.

In previous studies at FBV, avoidance of CNSL is reported to be the main causal factor to explain the lack of capuchin tool use to open fresh nuts at that site, with rubbing of the nut on hard substrate being favoured^25^,^36^. It appears reasonable, therefore, to propose that tool selection for cashew nut processing at SCNP may be affected by the caustic nature of fresh nuts. The use of larger hammers may help avoid contact with this hazard, both because it leads to fewer hits per nut (and so a reduced opportunity to come into contact with CNSL) and because the larger area of the tool may act to shield the monkey from the released liquid. The decreased risk of contact with caustic material from using a heavier (and therefore larger) pounding tool may outweigh any increased energetic costs.

The SCNP capuchins demonstrate a different, technology-based approach to the same hazard encountered by the FBV monkeys when feeding on fresh cashew nuts. In contrast to FBV, the SCNP capuchins have never been observed using a rubbing technique to open fresh cashew nuts^32^. We can, therefore, be relatively confident that the differences in fresh cashew processing seen at FBV and SCNP are not the product of incomplete observation at either site, and represent instead current, group-level, intra-species behavioural variation.

The origins of the behavioural diversity between populations remain to be investigated in more detail. Multiple driving factors, including genetic predispositions, environmental conditions and/or social learning mechanisms, could have led to the emergence of the observed differences. The species of capuchin at FBV and SCNP is the same (*S. libidinosus*), suggesting a genetic explanation for the distinct responses to fresh cashews at these sites is unlikely but cannot fully be excluded. Ecological circumstances will have to be directly compared between sites.

Cashew nuts appear to have similar caustic properties at both sites, as group members of each population shows chemical burns after direct contact with CNSL. Capuchins at each site have developed a group specific technique to avoid direct contact. We are not yet able to quantify the costs and benefits accrued to the capuchins using either the rubbing or stone pounding strategies to open fresh cashews. In both regions of Brazil the same two subspecies of cashew tress (*Anacardium occidentale* and *Anacardium microcarpum*) are present. However, the abundance of each species as well as the frequencies of which species is consumed by the capuchins is still unknown and no quantitative data from FBV on levels of CNSL are available.

Ecological circumstances at SCNP may favor the increased amount of stone tool use. Although the forms that stone tool use may take among the SCNP capuchins were unpredictable in advance, we should perhaps not be surprised that they have adopted a technological solution to the problem of fresh cashew processing. The SCNP capuchins have a more extensive and varied tool repertoire of stone and stick use, including using stone tools for digging, communication, and pulverizing other stones^46^,^47^,^48^. FBV capuchins in contrast use stone tools almost exclusively for pounding open hard palm nuts, a behaviour that they appear to have extended to dry, hard cashews but not the softer fresh cashew nuts.

Diversity between populations however, can also emerge through different social transmission pathways^49^,^50^. At this stage of our knowledge about cultural variation among animals such as primates, cetaceans, birds and fish^49^,^51^,^52^,^53^, this possibility cannot be neglected. Preliminary observations suggest that young individuals at SCNP are more interested in cashew processing by adult group members than was suggested for the FBV group^36^. Future comparative research will provide specific answers and detailed insight into factors responsible for behavioural diversity in wild capuchin populations.

The findings of this study suggest that capuchin monkeys may use stone tools not only merely to access encased food items, but for protective strategies to shield themselves from caustic liquids during food processing. Although requiring further investigation, a protective function of stone tools has not been previously reported for wild capuchin monkeys or any stone tool using non-human primates. The use of protective tools in the animal kingdom is rare and our study expands the notion to lithic technologies, providing a base for further research into the use of foraging tools and risk avoidance in wild animals.

## Additional Information

**How to cite this article**: Luncz, L. V. *et al.* Wild capuchin monkeys adjust stone tools according to changing nut properties. *Sci. Rep.* **6**, 33089; doi: 10.1038/srep33089 (2016).

## Supplementary Material

Supplementary Video 1

Supplementary Information

## Figures and Tables

**Table 1:** Predictions of cashew nut properties and capuchin processing behaviour at SCNP, Brazil.

Predictions	Results
Physical properties of cashew nuts	
a. CNSL decreases with increasing nut ripeness	Supported
b. Dry nuts are harder than fresh nuts	Supported
Capuchin behaviour	
a. Capuchins use fewer strikes when using a heavy stone	Supported
b. Capuchins select heavier stones for dry nuts	Rejected
