# Supplementary material for: Wild capuchin monkeys adjust stone tools according to changing nut properties
Source: Sci Rep. 2016 Sep 14;6:33089. doi: 10.1038/srep33089 (PMC5021971; doi:10.1038/srep33089)
Supplement: Supplementary Information [file srep33089-s2.doc]

Supplementary material for:

**Wild capuchin monkeys adjust stone tools according to changing nut properties**

Lydia V. Luncz^a^, Tiago Falótico^a,b^, Alejandra Pascual-Garrido^a^, Clara Corat^b^, Hannah Mosley^a^ and Michael Haslam^a^

^a^School of Archaeology, University of Oxford, UK

^b^Institute of Psychology, University of São Paulo, Brazil

Supplementary Tables:

|  | 2013 | 2014 |
| --- | --- | --- |
| Adult males | 8 | 7 |
| Adult females | 10 | 12 |
| Subadults | 2 | 2 |
| Juveniles | 11 | 8 |
| Infants | 4 | 7 |

Table S1. Composition of the Pedra Furada capuchin group in the years 2013 and 2014.

|  | Estimate | Std. Error | Z value | P value |
| --- | --- | --- | --- | --- |
| Intercept | 2.06 | 0.14 | 14.87 | <0.001 |
| Cashew stage (fresh) | 0.06 | 0.08 | 0.69 | 0.49 |
| Sex (male) | -0.19 | 0.16 | -1.22 | 0.22 |
| Tool weight | -0.19 | 0.04 | -4.82 | <0.001 |

Table S2. Results of a GLMM on the effects of various predictors on hammer weight selection.

|  | Estimate | Std. Error | Z value | P value |
| --- | --- | --- | --- | --- |
| Intercept | 0.89 | 0.06 | 14.7 | <0.001 |
| Cashew (fresh) | 0.30 | 0.06 | 5.05 | <0.001 |
| Sex (male) | 0.17 | 0.07 | 2.51 | 0.01 |

Table S3. Results of a GLMM on the effects of cashew ripeness stage on hammer weight selection.

Supplementary Video

Video 1: Wild capuchin monkeys *(Sapajus libidinosus)* participating in cashew nut cracking experiments at Serra da Capivara National Park.
